# Association between negative psychology and sleep quality in dialysis patients during the COVID‐19 pandemic

**DOI:** 10.1002/nop2.1681

**Published:** 2023-02-20

**Authors:** Liuyan Huang, Fan Zhang, Rong Zhu, Liya Wang, Yue Zhang, Huachun Zhang, Yifei Zhong

**Affiliations:** ^1^ Department of Nephrology Longhua Hospital Shanghai University of Traditional Chinese Medicine Shanghai China; ^2^ Blood Purification Center Longhua Hospital Shanghai University of Traditional Chinese Medicine Shanghai China; ^3^ Department of Nephrology Shanghai Tenth People's Hospital of Tongji University Shanghai China; ^4^ Department of Nursing Tongji Hospital of Tongji University Shanghai China; ^5^ Department of Nursing Longhua Hospital Shanghai University of Traditional Chinese Medicine Shanghai China

**Keywords:** anxiety, COVID‐19, depression, dialysis, negative psychology, sleep quality

## Abstract

**Aims and Objectives:**

The aim of this study was to assess the sleep quality in dialysis patients during the COVID‐19 epidemic and explore the association between negative psychology (including depression, anxiety, and stress) and sleep quality in this population.

**Design:**

A cross‐sectional study including three centres.

**Methods (Patients or Public Contribution):**

This cross‐sectional study included 378 dialysis patients from April to May 2022 in three dialysis centres in Shanghai. Methods. Depression, anxiety, stress, and sleep quality were measured by the Hospital Anxiety and Depression Scale (HADS), Perceived Stress Scale‐14 (PSS‐14), and Pittsburgh sleep quality index (PSQI), respectively. With a threshold of 5 to classify participants into good and poor sleep quality, with HADS/PSS‐14 scores as independent variables (per standard deviation (SD) increment), respectively and binary Logistic regression model was constructed to explore the association between the three negative psychological aspects of depression, anxiety, and stress and sleep quality.

**Results:**

The median PSQI score was 11.0 (mean ± SD: 11.8 ± 4.8). Among them, poor sleep quality (i.e., PSQI >5) was reported by 90.2% of participants. After adjusting for sociodemographic and disease‐related information, HADS‐depression was associated with a significant 49% (odds ratio (OR): 1.49; 95% CI 1.02–2.18) increase in the risk of poor sleep quality for each additional SD (2.4). Correspondingly, for each SD (7.1) increase in PSS‐14, the risk of poor sleep quality was significantly increased by 95% (OR: 1.95; 95% CI 1.35–2.82).

**Conclusion:**

During the COVID‐19 pandemic, there was a significant negative association between negative psychology, such as depression and stress, and sleep quality in dialysis patients, and this relationship was independent of the dialysis modality.

**Relevance to Clinical Practice:**

In the context of the rampant COVID‐19, the vast majority of dialysis‐dependent chronic kidney disease presents with severe sleep quality problems, and negative psychology is a potential influencing factor.

## 1 INTRODUCTION

2019 Coronavirus (COVID‐19) is a highly infectious virus that can cause severe respiratory distress in humans (Zhu et al., 2020). In March 2020, the World Health Organization declared COVID‐19 a global pandemic due to a rapid virus outbreak (WHO, 2020). In March 2022, COVID‐19 swept through Shanghai, China. In response, the Shanghai government announced drastic measures: keeping social distance such as working from home and closing all public transportation, restaurants, and sports facilities to limit the further spread of the virus (Zhang et al., 2022). This initiative posed a significant challenge to the lives and medical care of the city's 25 million residents, dialysis‐dependent CKD patients are one of the more severely affected populations in this context.

The renal community is increasingly aware of the high symptom burden and impaired quality of life experienced by patients with chronic kidney disease (Fletcher et al., 2022). There has also been a recent shift in the focus of research, with patient‐reported outcomes, including sleep quality, being advocated as a focus of attention in chronic kidney disease, particularly in dialysis‐dependent populations (Tong et al., 2017). Among the many symptoms experienced in CKD, poor sleep quality is one of the most commonly reported symptoms (Natale et al., 2019).

During a COVID‐19 pandemic, the restrictions of social life, isolation, lack of information, and fear of the virus can lead to various negative psychological aspects in the general population (Xiang et al., 2020). Patients with chronic kidney disease treated with dialysis are at higher risk of COVID‐19 infection and have a worse prognosis (Corbett et al., 2020; Rombolà & Brunini, 2020). In this context, the already fragile bodies of dialysis patients combined with the negative psychology of anxiety, depression, and stress brought about by the epidemic have led to deteriorating sleep quality in this population (Bonenkamp et al., 2021; Hao et al., 2021; Nadort et al., 2022).

In order to develop interventions targeting sleep quality in this population, it is first necessary to understand the factors associated with poor sleep quality, and one of the modifiable factors may be negative psychology, which refers to negative emotional states such as anxiety, depression, stress, and nervousness (Singh et al., 2008). Considering that Shanghai has just experienced a “war” with COVID‐19 (Taylor, 2022), this study was conducted to investigate the association between negative psychology and sleep quality of dialysis patients in the context of the epidemic. More specifically, we analysed how anxiety, depression, and stress affect the quality of sleep in dialysis patients.

### 1.1 Methods

The present report follows the Strengthening the Reporting of Observational Studies in Epidemiology (STROBE) (von Elm et al., 2008) statement (Table S1) for cross‐sectional studies.

### 1.2 Sampling and sample size

To ensure sample heterogeneity and data richness, purposive sampling was used in selecting subjects. The PSQI has a total of 19 items. According to the sample size requirement for regression analysis, the ideal sample size should be 5–10 times the number of entries, and considering the absence of response bias, the sample size should be increased by 10%. Therefore, the ideal sample size = 19*(5–10)*(1 + 10%) = 105 ~ 209. There should be adequate power since the actual sample size (n = 378) was far more than the required sample size.

Inclusion criteria included (i) chronic kidney disease patients receiving dialysis treatment (including peritoneal dialysis and haemodialysis), (ii) long‐term residence in the Shanghai area, and (iii) signing an online informed consent form. Data with incomplete information were excluded.

### 1.3 Ethical considerations

The Ethics Committee approved the ethical approval for this cross‐sectional study of Longhua Hospital Shanghai University of Traditional Chinese Medicine (permit number: 2022LCSY021). All participants have given informed consent prior to the study.

### 1.4 Data collection

A cross‐sectional survey of dialysis‐dependent chronic kidney disease patients from three centres (Longhua Hospital Shanghai University of Traditional Chinese Medicine, Shanghai Tenth People's Hospital of Tongji University, and Tongji Hospital of Tongji University) was conducted during the Shanghai COVID‐19 pandemic. Data were collected between April and May 2022 using a self‐reported online survey (Questionnaire Star: www.wjx.cn). Questionnaire items were developed on the platform and then shared with individuals to collect data in an isolated setting. The entire questionnaire took approximately 20 minutes to complete.

### 1.5 Study instrument

General information includes sociodemographic information (gender, age, and education) and disease‐related information (body mass index, dialysis modality, dialysis vintage, and comorbidities).

The Pittsburgh Sleep Quality Index (PSQI) measured subjective sleep quality over the past month (Buysse et al., 1989). The PSQI consists of 19 self‐assessment questions divided into seven component scores. Each score is equally weighted on a scale of 0–3. The seven component scores are summed to provide a total PSQI score (ranging from 0 to 21). A global score of 5 or more indicates poor sleep quality: the higher the score, the worse the sleep quality (Buysse et al., 1989). The Chinese version of the PSQI was translated by Tsai et al. (Tsai et al., 2005) and has been tested with good reliability and validity. The Cronbach's α coefficient of the PSQI in this study was 0.75.

Anxiety and depression were measured by a Chinese version of the Hospital Anxiety and Depression Scale (HADS), developed by Zigmond and Snaith (Zigmond & Snaith, 1983). The HADS is a 14‐item, 4‐point scale (from 0 to 3) consisting of an anxiety scale (seven items, HADS‐A) and a depression scale (seven items, HADS‐D); scores for each domain range from 0 to 21 (Bjelland et al., 2002). Higher scores indicate higher levels of anxiety or depression. The HADS has been validated in China population with Cronbach's α of 0.83 (HADS‐A) and 0.82 (HADS‐D) (Lam et al., 1995).

The Perceived Stress Scale‐14 (PSS‐14) is a 14‐item self‐report scale to assess “how one sees common life situations as stressful over the last month,” i.e., self‐perceived stress (Leung et al., 2010). As an overall measure of stress levels, respondents were asked to report whether their lives were unpredictable, uncontrollable, or overloaded (Cohen et al., 1983). Each item is rated on a 5‐point scale (0 = never, 1 = almost never, 2 = sometimes, 3 = often, 4 = very often), with six positive and eight negative items. In this study, the Cronbach's α of PSS‐14 Chinese version was 0.880, indicating that it had good internal consistency reliability.

### 1.6 Statistical analysis

As the PSQI was skewed, non‐parametric tests were used to compare scores for different sociodemographic characteristics. When the non‐parametric Kruskal‐Wallis test showed significance, a Dunn's *t*‐test was used for post‐hoc tests. To indicate the precision of differences, we also calculated mean differences (MD) and 95% confidence intervals (CI). Spearman's correlation coefficients were used to test correlations between continuous variables (HADS‐A/HADS‐D/PSS‐14 and PSQI). To determine the association of anxiety, depression, and stress with sleep quality, we developed binary logistic regression models using a cut‐off of 5 to classify participants as having good sleep quality (PSQI ≤5) and poor sleep quality (PSQI >5), with HADS‐A/HADS‐D/PSS scores as independent variables (per standard deviation (SD) increment), respectively. We fitted three logistic regression models: model 1 was unadjusted, model 2 included only demographic information, and model 3 had sociodemographic and disease‐related information. Next, we performed a subgroup analysis of logistic regression models for dialysis type. The significance level for all comparisons was 0.05.

## 2 RESULTS

### 2.1 Sociodemographic and disease‐related characteristics of the participants

Finally, all participants completed the questionnaire (response rate = 100%). Of the 378 participants, more than half were 60 years of age or older; there was little difference between males and females; 197 participants had an education level of college and above. In addition, 202 participants had a normal BMI; 230 were patients with peritoneal dialysis‐dependent CKD; approximately half of the participants had been on dialysis for <3 years, and only 6.6% had no comorbidities (Table 1).

**TABLE 1:** Characteristics of the 378 participants included in the study.

Variable	Overall (%)	PSQI	MD (95% CI)	*p*‐Value
All		11.0 (8.0, 16.0)		
Age group				
<60 years	153 (40.5%)	10.0 (7.0, 13.0)	−2.5 (−3.5, −1.5)	<0.001
≥60 years	225 (59.5%)	13.0 (9.0, 17.0)		
Gender				
Male	203 (53.7%)	11.0 (8.0, 15.0)	−0.3 (−1.3, 0.7)	0.530
Female	175 (46.3%)	12.0 (8.0, 16.0)		
Education				
Below high school	181 (47.9%)	12.0 (8.5, 16.0)	0.6 (−0.4, 1.6)	0.256
College and above	197 (52.1%)	11.0 (8.0, 15.0)		
BMI (kg/m^2^)				
Lean (≤18.4)	45 (11.9%)	11.0 (7.0, 15.5)	−0.4 (−2.0, 1.1)	0.463
Normal (18.5–23.9)	202 (53.4%)	11.0 (8.0, 16.0)	−0.4 (−1.5, 0.6)	
Overweight (≥24.0)	131 (34.7%)	12.0 (9.0, 16.0)		
Dialysis modality			−1.1 (−2.1, −0.1)	0.028
Peritoneal dialysis	230 (60.8%)	11.0 (8.0, 15.0)		
Haemodialysis	148 (39.2%)	12.0 (9.0, 17.0)		
Dialysis vintage (years)				
<3	179 (47.4%)	11.0 (8.0, 15.0)	−2.0 (−3.1, −0.8)	0.001
3 ~ 5	89 (23.5%)	12.0 (8.5, 15.0)	−1.5 (−2.9, −0.2)	0.05
>5	110 (29.1%)	14.0 (9.0, 17.3)		
Comorbidity				
None	25 (6.6%)	9.0 (7.0, 11.0)	−3.5 (−5.5, −1.6)	<0.001
One	137 (36.2%)	11.0 (7.0, 15.0)	−1.9 (−2.9, −0.9)	0.001
More than one	216 (57.1%)	12.5 (9.0, 17.0)		

Abbreviations: CI, confident interval; MD, mean difference; PSQI, Pittsburgh Sleep Quality Index.

### 2.2 Sleep quality of dialysis patients

**FIGURE 1:**
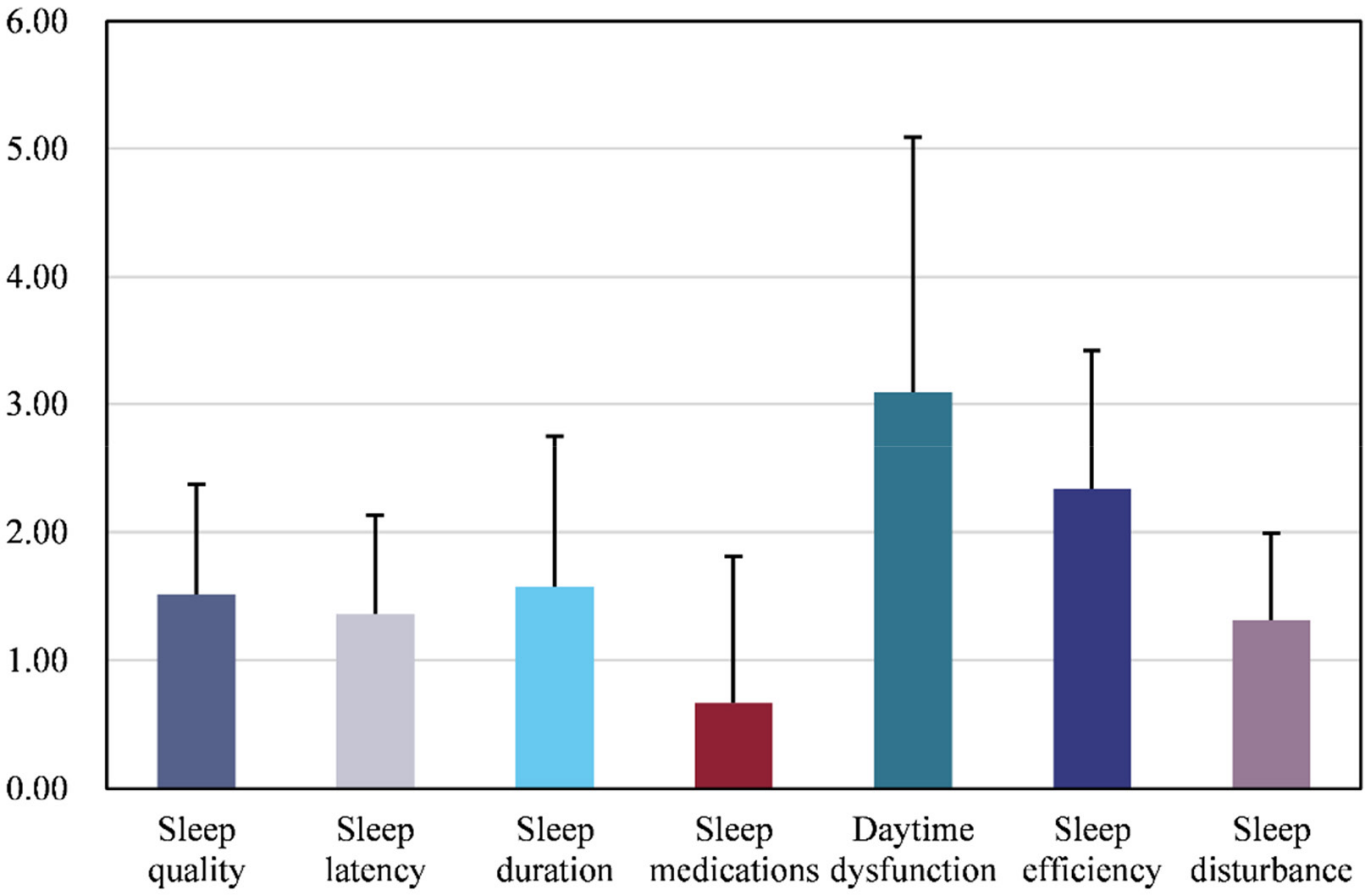
PSQI scores on seven dimensions.

The median PSQI score was 11.0 (mean ± SD: 11.8 ± 4.8). Among them, Poor sleep quality (i.e., PSQI >5) was reported by 90.2% of participants. PSQI scores were significantly higher in patients over 60 years of age than in those below 60 years. PSQI scores were substantially higher in haemodialysis‐dependent CKD patients than in peritoneal dialysis‐dependent ones. The PSQI scores were higher in patients with longer dialysis vintage and more comorbidities (Table 1 **)**. Scores for the seven dimensions of the PSQI scale are shown in Figure 1, where daytime dysfunction scores were the highest.

### 2.3 Correlations between HADS‐A/HADS‐D/PSS‐14 and PSQI

**FIGURE 2:**
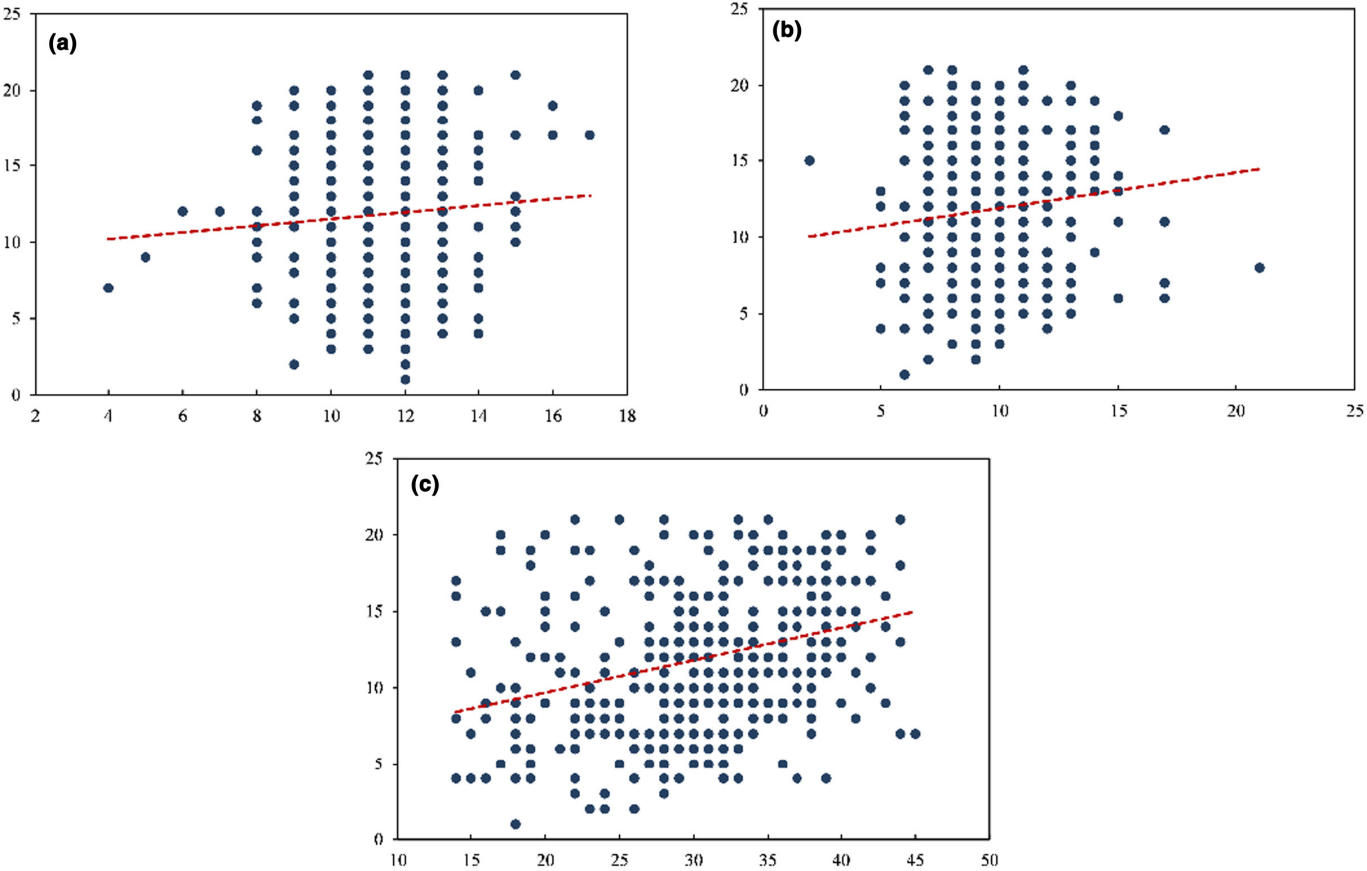
Scatterplot of negative psychological scales and PSQI scores (a) HADS‐A and PSQI; (b) HADS‐D and PSQI; (c) PSS‐14 and PSQI.

The Spearman correlation analysis revealed that HADS‐A, HADS‐D, and PSS‐14 scores were all positively correlated with PSQI with correlation coefficients of *r* = 0.035 (*p* = 0.495), *r* = 0.124 (*p* = 0.016), *r* = 0.325 (*p* < 0.001), respectively (Figure 2).

### 2.4 Binary logistic regression models

After adjusting for sociodemographic and disease‐related information, each SD increase in HADS‐A (1.6) was associated with a 29% (95% CI 0.56–1.18) reduction in the risk of poor sleep quality, but this difference was not statistically significant. From a depression and stress perspective, HADS‐D was associated with a significant 49% (95% CI 1.02–2.18) increase in the risk of poor sleep quality for each additional SD (2.4). Correspondingly, for each SD (7.1) increase in PSS, the risk of poor sleep quality was significantly increased by 95% (95% CI 1.35–2.82) (Table 2).

**TABLE 2:** Binary logistic regression models for the association between negative psychology and sleep quality.

	Model 1	Model 2	Model 3
Anxiety			
Per SD increase	0.81 (0.57, 1.15)	0.78 (0.55, 1.12)	0.81 (0.56, 1.18)
Depression			
Per SD increase	1.51 (1.03, 2.20)	1.48 (1.00, 2.18)	1.49 (1.02, 2.18)
Stress			
Per SD increase	2.00 (1.41, 2.85)	2.01 (1.41, 2.88)	1.95 (1.35, 2.82)

*Note*: Data are presented as an odds ratio (95% CI). Model 1 Unadjusted. Model 2 Adjusted for age, gender, and education. Model 3 Adjusted for age, gender, education, body mass index, dialysis modality, dialysis vintage, and comorbidity.

Abbreviation: SD, standard deviation.

### 2.5 Subgroup analysis based on dialysis model

We performed a subgroup analysis stratifying the association between negative psychology and sleep quality by dialysis model, as shown in Table 3. There were no interactions between peritoneal dialysis and haemodialysis and the association between negative psychology and sleep quality. The positive relationship between HADS‐D, PSS‐14, and PSQI remained consistent among participants in both dialysis modalities (Table 3).

**TABLE 3:** Multivariable adjusted OR for the association between negative psychology and sleep quality by dialysis modality.

	Model 1	Model 2	Model 3	*p*_for interaction_
Anxiety				0.418
Peritoneal dialysis	0.87 (0.58, 1.31)	0.82 (0.53, 1.26)	0.93 (0.59, 1.47)	
Haemodialysis	0.69 (0.35, 1.35)	0.70 (0.35, 1.38)	0.72 (0.37, 1.42)	
Depression				0.175
Peritoneal dialysis	1.72 (1.10, 2.69)	1.71 (1.08, 2.71)	1.79 (1.13, 2.84)	
Haemodialysis	1.01 (0.49, 2.09)	1.04 (0.48, 2.24)	1.03 (0.47, 2.27)	
Stress				0.192
Peritoneal dialysis	1.73 (1.14, 2.64)	1.72 (1.12, 2.62)	1.68 (1.07, 2.63)	
Haemodialysis	2.64 (1.35, 5.14)	2.83 (1.39, 5.73)	2.87 (1.40, 5.91)	

*Note*: Data are presented as an odds ratio (95% CI). Model 1 Unadjusted. Model 2 Adjusted for age, gender, and education. Model 3 Adjusted for age, gender, education, body mass index, dialysis vintage, comorbidity.

## 3 DISCUSSION

This study showed that 90.2% of dialysis patients reported poor sleep quality (i.e., PSQI>5) as measured by the PSQI scale. In addition, high levels of depression and stress were significantly associated with poor sleep quality after adjusting for demographic and disease‐related information. Quite unexpectedly, anxiety favoured improved sleep quality as a protective factor. Moreover, the above associations were independent of the dialysis modality of the participants.

The sleep quality of dialysis patients usually decreases, and sleep disturbances are often present even in the early stages of kidney disease. In dialysis patients, the prevalence of any sleep disorder ranges from 45% to 80% (Iliescu et al., 2004). The COVID‐19 outbreak was a new and highly evolved stressor for all susceptible populations, including chronically ill and dialysis patients, due to daily life, social isolation, and public transportation disruptions. Al Naamani et al. (Al Naamani et al., 2021) reported that 56.9% of haemodialysis patients reported poor sleep quality during the COVID‐19 pandemic. Correspondingly, poor sleep quality was higher in the dialysis patients included in this study. We attributed this to three reasons: (i) it is well known that haemodialysis patients usually must travel weekly to healthcare facilities for regular treatment (Uchida et al., 2022). However, although traffic blocking is an important strategy to prevent the spread of the COVID‐19 virus (Jombart et al., 2011), it causes great inconvenience to haemodialysis patients in their travels. The resulting tension is a crucial factor contributing to poor sleep quality (De Silva et al., 2021). (ii) More than half of the subjects in this survey were elderly, and advanced age was associated with an increased likelihood of sleep difficulties (Viola et al., 2012; Zhang et al., 2021). (iii) comorbidities such as restless legs syndrome and dialysis adequacy in patients with kidney disease are important contributors to the severity of sleep problems in this population (Kalantar‐Zadeh et al., 2022; Örsal et al., 2017).

In this investigation, depression and perceived stress were prominent contributors to poor sleep quality among dialysis patients. In the COVID‐19 outbreak, haemodialysis patients travel between home and healthcare facilities (Yu et al., 2021), are at constant risk of contracting the virus, and are under tremendous pressure on their health and their families (Bulbul et al., 2022; Lv et al., 2022). In addition, loneliness, fear of death, and financial concerns are stressors that lead to anxiety and depression during the COVID‐19 pandemic, and the poor sleep quality caused by such stress is expected (Duru, 2022). As the results of this study show, every 7‐point increase in the PSS‐14 scale is associated with a 95% increased risk of poor sleep quality, which can be devastating for the prognosis of dialysis patients. On the other hand, the COVID‐19 epidemic suddenly, uncontrollably, and unpredictably. The psychological panic and depression and economic income blockage caused in the short term can cause greater or lesser psychological damage to individuals (Lee et al., 2018), especially those with chronic diseases. They may predispose to depressive symptoms (Mushtaque et al., 2022). Studies including dialysis patients have shown an association between depression and poor sleep quality (Liaveri et al., 2017; Trbojević‐Stanković et al., 2014), and the present study reveals the same results: with each 2.4‐point increase in the HADS‐D scale, the risk of poor sleep quality increased by 49%. Surprisingly, in this study, anxiety seemed to contribute to sleep quality. This result explains that (i) the conversion of PSQI scores to categorical variables may have changed the relationship between the variables. As can be obtained from the scatter plot, HADS‐A was positively correlated with PSQI, i.e., anxiety was negatively correlated with sleep quality. (ii) The study did not perform a sample size calculation, weakening the statistical power.

In subgroup analysis, we found that the adverse association between negative psychology and sleep quality was consistent in peritoneal dialysis and haemodialysis patients. Interestingly, depression significantly impacted sleep quality in peritoneal dialysis patients, while stress significantly impacted sleep quality in haemodialysis patients.

Based on this finding and the potential for continued pressure from the COVID‐19 epidemic, we propose the following recommendations: (i) Given the high prevalence of poor sleep quality in dialysis patients, healthcare workers in dialysis centres should develop appropriate non‐pharmacological therapies to alleviate this phenomenon. Some studies have shown that mindfulness meditation improves sleep quality in the general population and COIVD‐19 patients (Desai et al., 2021; Hausswirth et al., 2022; Li et al., 2022). (ii) Policymakers should focus on the psychological status of the chronically ill population, who, after all, have to deal with both physical and psychological stress caused by COVID‐19 (Bonenkamp et al., 2021; Urquhart‐Secord et al., 2016).

Although this study collected dialysis patients from multiple centres, some limitations need to be noted. First, an a priori sample size calculation was not performed. Second, this investigation was limited to dialysis‐dependent patients with chronic kidney disease, and the generalizability to the pre‐dialysis and renal transplant recipient populations needs to be further validated. Third, this study only collected information during the COVID‐19 epidemic, and longitudinal studies are required to investigate how negative psychology alters sleep quality in dialysis patients.

## 4 CONCLUSION

During the COVID‐19 pandemic, there was a significant negative association between negative psychology, such as depression and stress, and sleep quality in dialysis patients. This relationship was independent of the dialysis modality.

## AUTHOR CONTRIBUTIONS

Research idea and study design: Liuyang Huang and Huachun Zhang. Data collection: Fan Zhang, Liuyan Huang, Rong Zhu, Yue Zhang, and Liya Wang. Data analysis/interpretation: Fan Zhang. Manuscript drafting: Fan Zhang. Editing and revising: Liuyan Huang and Yifei Zhong. All authors have approved the submitted version and agreed to be accountable for the author's own contributions.

## FUNDING INFORMATION

Municipal Human Resources Development Program for Outstanding Leaders in Medical Disciplines in Shanghai (2017BR023).

## CONFLICT OF INTEREST STATEMENT

The authors declare that they have no competing interests.

## RESEARCH ETHICS COMMITTEE APPROVAL

The Ethics Committee approved the ethical approval for this cross‐sectional study of Longhua Hospital, Shanghai University of Traditional Chinese Medicine, and no ethical consent was required. All participants provided written informed consent in this study.

## Supporting information


Table S1.

## Data Availability

All data generated or analysed during this study are included in this published article.

## References

[nop21681-bib-0001] Al Naamani, Z. , Gormley, K. , Noble, H. , Santin, O. , & Al Maqbali, M. (2021). Fatigue, anxiety, depression and sleep quality in patients undergoing haemodialysis. BMC Nephrology, 22(1), 157. 10.1186/s12882-021-02349-3 33910523 PMC8080199

[nop21681-bib-0002] Bjelland, I. , Dahl, A. A. , Haug, T. T. , & Neckelmann, D. (2002). The validity of the hospital anxiety and depression scale. An updated literature review. Journal of Psychosomatic Research, 52(2), 69–77. 10.1016/s0022-3999(01)00296-3 11832252

[nop21681-bib-0003] Bonenkamp, A. A. , Druiventak, T. A. , Van Eck Van der Sluijs, A. , Van Ittersum, F. J. , Van Jaarsveld, B. C. , & Abrahams, A. C. (2021). The impact of COVID‐19 on the mental health of dialysis patients. Journal of Nephrology, 34(2), 337–344. 10.1007/s40620-021-01005-1 33742413 PMC7978448

[nop21681-bib-0004] Bulbul, E. , Dogan, P. , Sendir, M. , Kaya, A. , & Ozdemir, C. (2022). Determination of problems experienced during the COVID‐19 pandemic by individuals receiving hemodialysis treatment. Hemodialysis International, 26(1), 74–82. 10.1111/hdi.12960 34196085

[nop21681-bib-0005] Buysse, D. J. , Reynolds, C. F., 3rd , Monk, T. H. , Berman, S. R. , & Kupfer, D. J. (1989). The Pittsburgh sleep quality index: A new instrument for psychiatric practice and research. Psychiatry Research, 28(2), 193–213. 10.1016/0165-1781(89)90047-4 2748771

[nop21681-bib-0006] Cohen, S. , Kamarck, T. , & Mermelstein, R. (1983). A global measure of perceived stress. Journal of Health and Social Behavior, 24(4), 385–396. 6668417

[nop21681-bib-0007] Corbett, R. W. , Blakey, S. , Nitsch, D. , Loucaidou, M. , McLean, A. , Duncan, N. , & Ashby, D. R. (2020). Epidemiology of COVID‐19 in an urban dialysis center. Journal of the American Society of Nephrology, 31(8), 1815–1823. 10.1681/ASN.2020040534 32561681 PMC7460899

[nop21681-bib-0008] De Silva, I. , Evangelidis, N. , Hanson, C. S. , Manera, K. , Guha, C. , Scholes‐Robertson, N. , & Tong, A. (2021). Patient and caregiver perspectives on sleep in dialysis. Journal of Sleep Research, 30(4), e13221. 10.1111/jsr.13221 33103303

[nop21681-bib-0009] Desai, K. , Gupta, P. , Parikh, P. , & Desai, A. (2021). Impact of virtual Heartfulness meditation program on stress, quality of sleep, and psychological wellbeing during the COVID‐19 pandemic: A mixed‐method study. International Journal of Environmental Research and Public Health, 18(21), 111114. 10.3390/ijerph182111114 PMC8583339 34769634

[nop21681-bib-0010] Duru, H. (2022). The prevalence and severity of mental health problems and sexual dysfunction in hemodialysis patients before and during the COVID‐19 pandemic. Therapeutic Apheresis and Dialysis, 26, 1211–1219. 10.1111/1744-9987.13805 35088541

[nop21681-bib-0011] Fletcher, B. R. , Damery, S. , Aiyegbusi, O. L. , Anderson, N. , Calvert, M. , Cockwell, P. , Ferguson, J. , Horton, M. , Paap, M. C. S. , Sidey‐Gibbons, C. , Slade, A. , Turner, N. , & Kyte, D. (2022). Symptom burden and health‐related quality of life in chronic kidney disease: A global systematic review and meta‐analysis. PLoS Medicine, 19(4), e1003954. 10.1371/journal.pmed.1003954 35385471 PMC8985967

[nop21681-bib-0012] Hao, W. , Tang, Q. , Huang, X. , Ao, L. , Wang, J. , & Xie, D. (2021). Analysis of the prevalence and influencing factors of depression and anxiety among maintenance dialysis patients during the COVID‐19 pandemic. International Urology and Nephrology, 53(7), 1453–1461. 10.1007/s11255-021-02791-0 33675473 PMC7936244

[nop21681-bib-0013] Hausswirth, C. , Nesi, X. , Dubois, A. , Duforez, F. , Rougier, Y. , & Slattery, K. (2022). Four weeks of a neuro‐meditation program improves sleep quality and reduces hypertension in nursing staff during the COVID‐19 pandemic: A parallel randomized controlled trial. Frontiers in Psychology, 13, 854474. 10.3389/fpsyg.2022.854474 35645851 PMC9130829

[nop21681-bib-0014] Iliescu, E. A. , Yeates, K. E. , & Holland, D. C. (2004). Quality of sleep in patients with chronic kidney disease. Nephrology, Dialysis, Transplantation, 19(1), 95–99. 10.1093/ndt/gfg423 14671044

[nop21681-bib-0015] Jombart, T. , Eggo, R. M. , Dodd, P. J. , & Balloux, F. (2011). Reconstructing disease outbreaks from genetic data: A graph approach. Heredity, 106(2), 383–390. 10.1038/hdy.2010.78 20551981 PMC3183872

[nop21681-bib-0016] Kalantar‐Zadeh, K. , Lockwood, M. B. , Rhee, C. M. , Tantisattamo, E. , Andreoli, S. , Balducci, A. , & Li, P. K. (2022). Patient‐centred approaches for the management of unpleasant symptoms in kidney disease. Nature Reviews. Nephrology, 18(3), 185–198. 10.1038/s41581-021-00518-z 34980890

[nop21681-bib-0017] Lam, C. L. , Pan, P. C. , Chan, A. W. , Chan, S. Y. , & Munro, C. (1995). Can the hospital anxiety and depression (HAD) scale be used on Chinese elderly in general practice? Family Practice, 12(2), 149–154. 10.1093/fampra/12.2.149 7589936

[nop21681-bib-0018] Lee, S. M. , Kang, W. S. , Cho, A. R. , Kim, T. , & Park, J. K. (2018). Psychological impact of the 2015 MERS outbreak on hospital workers and quarantined hemodialysis patients. Comprehensive Psychiatry, 87, 123–127. 10.1016/j.comppsych.2018.10.003 30343247 PMC7094631

[nop21681-bib-0019] Leung, D. Y. , Lam, T. H. , & Chan, S. S. (2010). Three versions of perceived stress scale: Validation in a sample of Chinese cardiac patients who smoke. BMC Public Health, 10, 513. 10.1186/1471-2458-10-513 20735860 PMC2939644

[nop21681-bib-0020] Li, J. , Zhang, Y. Y. , Cong, X. Y. , Ren, S. R. , Tu, X. M. , & Wu, J. F. (2022). 5‐min mindfulness audio induction alleviates psychological distress and sleep disorders in patients with COVID‐19. World Journal of Clinical Cases, 10(2), 576–584. 10.12998/wjcc.v10.i2.576 35097083 PMC8771375

[nop21681-bib-0021] Liaveri, P. G. , Dikeos, D. , Ilias, I. , Lygkoni, E. P. , Boletis, I. N. , Skalioti, C. , & Paparrigopoulos, T. (2017). Quality of sleep in renal transplant recipients and patients on hemodialysis. Journal of Psychosomatic Research, 93, 96–101. 10.1016/j.jpsychores.2016.12.013 28107900

[nop21681-bib-0022] Lv, H. , Meng, J. , Chen, Y. , Yang, F. , Wang, W. , Wei, G. , Zhang, J. , Wang, H. , Wang, M. , Zhou, L. , & Liu, H. (2022). Impact of COVID‐19 pandemic on elevated anxiety symptoms of maintenance hemodialysis patients in China: A one‐year follow‐up study. Frontiers in Psychiatry, 13, 864727. 10.3389/fpsyt.2022.864727 35664473 PMC9160521

[nop21681-bib-0023] Mushtaque, I. , Awais, E. Y. M. , Zahra, R. , & Anas, M. (2022). Quality of life and illness acceptance among end‐stage renal disease (ESRD) patients on hemodialysis: The moderating effect of death anxiety during COVID‐19 pandemic. Omega, 302228221075202. Advance online publication., 003022282210752. 10.1177/00302228221075202 PMC8902316 35254867

[nop21681-bib-0024] Nadort, E. , Rijkers, N. , Schouten, R. W. , Hoogeveen, E. K. , Bos, W. J. W. , Vleming, L. J. , Westerman, M. , Schouten, M. , MJE, D. , YFC, S. , Shaw, P. C. , Farhat, K. , Dekker, F. W. , van Oppen, P. , CEH, S. , & Broekman, B. F. P. (2022). Depression, anxiety and quality of life of hemodialysis patients before and during the COVID‐19 pandemic. Journal of Psychosomatic Research, 158, 110917. 10.1016/j.jpsychores.2022.110917 35462121 PMC9008976

[nop21681-bib-0025] Natale, P. , Ruospo, M. , Saglimbene, V. M. , Palmer, S. C. , & Strippoli, G. F. (2019). Interventions for improving sleep quality in people with chronic kidney disease. *The* . Cochrane Database of Systematic Reviews, 5(5), CD012625. 10.1002/14651858.CD012625.pub2 31129916 PMC6535156

[nop21681-bib-0026] Örsal, Ö. , Ünsal, A. , Balcı‐Alparslan, G. , & Duru, P. (2017). Restless legs syndrome and sleep quality in patients on hemodialysis. Nephrology Nursing Journal, 44(2), 167–176. 29165968

[nop21681-bib-0027] Rombolà, G. , & Brunini, F. (2020). COVID‐19 and dialysis: Why we should be worried. Journal of Nephrology, 33(3), 401–403. 10.1007/s40620-020-00737-w 32323202 PMC7175820

[nop21681-bib-0028] Singh, K. , & Jha, S. D. (2008). Positive and negative affect, and grit as predictors of happiness and life satisfaction. Journal of the Indian Academy of Applied Psychology., 34, 40–45.

[nop21681-bib-0029] Taylor, L. (2022). Covid‐19: China installs fences and alarms in Shanghai in effort to curb cases. BMJ British Medical Journal (Clinical Research Ed.), 377, o1076. 10.1136/bmj.o1076 35477680

[nop21681-bib-0030] Tong, A. , Manns, B. , Hemmelgarn, B. , Wheeler, D. C. , Evangelidis, N. , Tugwell, P. , Crowe, S. , Van Biesen, W. , Winkelmayer, W. C. , O'Donoghue, D. , Tam‐Tham, H. , Shen, J. I. , Pinter, J. , Larkins, N. , Youssouf, S. , Mandayam, S. , Ju, A. , Craig, J. C. , & Investigators, S. O. N. G.‐H. D. (2017). Establishing Core outcome domains in hemodialysis: Report of the standardized outcomes in nephrology‐hemodialysis (SONG‐HD) consensus workshop. American Journal of Kidney Diseases, 69(1), 97–107. 10.1053/j.ajkd.2016.05.022 27497527 PMC5369351

[nop21681-bib-0031] Trbojević‐Stanković, J. , Stojimirović, B. , Bukumirić, Z. , Hadzibulić, E. , Andrić, B. , Djordjević, V. , & Jovanović, D. (2014). Depression and quality of sleep in maintenance hemodialysis patients. Srpski Arhiv za Celokupno Lekarstvo, 142(7–8), 437–443. 25233688

[nop21681-bib-0032] Tsai, P. S. , Wang, S. Y. , Wang, M. Y. , Su, C. T. , Yang, T. T. , Huang, C. J. , & Fang, S. C. (2005). Psychometric evaluation of the Chinese version of the Pittsburgh sleep quality index (CPSQI) in primary insomnia and control subjects. Quality of Life Research, 14(8), 1943–1952. 10.1007/s11136-005-4346-x 16155782

[nop21681-bib-0033] Uchida, J. , Yoshikoshi, S. , Nakajima, T. , Fukuzaki, N. , Suzuki, Y. , Yamamoto, S. , & Matsunaga, A. (2022). Impact of the COVID‐19 pandemic on depressive symptoms in Japanese patients undergoing hemodialysis. Journal of Nephrology, 35(1), 371–373. 10.1007/s40620-021-01218-4 34988940 PMC8731130

[nop21681-bib-0034] Urquhart‐Secord, R. , Craig, J. C. , Hemmelgarn, B. , Tam‐Tham, H. , Manns, B. , Howell, M. , & Tong, A. (2016). Patient and caregiver priorities for outcomes in hemodialysis: An international nominal group technique study. American Journal of Kidney Diseases, 68(3), 444–454. 10.1053/j.ajkd.2016.02.037 26968042

[nop21681-bib-0035] Viola, A. U. , Chellappa, S. L. , Archer, S. N. , Pugin, F. , Götz, T. , Dijk, D. J. , & Cajochen, C. (2012). Interindividual differences in circadian rhythmicity and sleep homeostasis in older people: Effect of a PER3 polymorphism. Neurobiology of Aging, 33(5), 1010.e17–1010.e1.01E27. 10.1016/j.neurobiolaging.2011.10.024 22169200

[nop21681-bib-0036] von Elm, E. , Altman, D. G. , Egger, M. , Pocock, S. J. , Gøtzsche, P. C. , Vandenbroucke, J. P. , & Initiative, S. T. R. O. B. E. (2008). The strengthening the reporting of observational studies in epidemiology (STROBE) statement: Guidelines for reporting observational studies. Journal of Clinical Epidemiology, 61(4), 344–349. 10.1016/j.jclinepi.2007.11.008 18313558

[nop21681-bib-0037] WHO . (2020). WHO Director‐General's opening remarks at the media briefing on COVID‐19 ‐ 18 March 2020. Retrieved from. https://www.who.int/director‐general/speeches/detail/who‐director‐general‐s‐opening‐remarks‐at‐the‐media‐briefing‐on‐covid‐19‐‐‐18‐march‐2020

[nop21681-bib-0038] Xiang, Y. T. , Yang, Y. , Li, W. , Zhang, L. , Zhang, Q. , Cheung, T. , & Ng, C. H. (2020). Timely mental health care for the 2019 novel coronavirus outbreak is urgently needed. The Lancet. Psychiatry, 7(3), 228–229. 10.1016/S2215-0366(20)30046-8 32032543 PMC7128153

[nop21681-bib-0039] Yu, J. Y. , Kim, J. S. , Hong, C. M. , Lee, K. Y. , Cho, N. J. , Park, S. , & Lee, E. Y. (2021). Psychological distress of patients with end‐stage kidney disease undergoing dialysis during the 2019 coronavirus disease pandemic: A cross‐sectional study in a university hospital. PLoS One, 16(12), e0260929. 10.1371/journal.pone.0260929 34860844 PMC8641873

[nop21681-bib-0040] Zhang, H. , Yang, Y. , Huang, J. , Lailan, S. , & Tao, X. (2021). Correlates of objective sleep quality in older peritoneal dialysis patients. Renal Failure, 43(1), 180–187. 10.1080/0886022X.2020.1871369 33459122 PMC7833042

[nop21681-bib-0041] Zhang, X. , Zhang, W. , & Chen, S. (2022). Shanghai's life‐saving efforts against the current omicron wave of the COVID‐19 pandemic. Lancet, 399(10340), 2011–2012. 10.1016/S0140-6736(22)00838-8 35533708 PMC9075855

[nop21681-bib-0042] Zhu, N. , Zhang, D. , Wang, W. , Li, X. , Yang, B. , Song, J. , Zhao, X. , Huang, B. , Shi, W. , Lu, R. , Niu, P. , Zhan, F. , Ma, X. , Wang, D. , Xu, W. , Wu, G. , Gao, G. F. , & Tan, W. (2020). A novel coronavirus from patients with pneumonia in China, 2019. The New England Journal of Medicine, 382(8), 727–733. 10.1056/NEJMoa2001017 31978945 PMC7092803

[nop21681-bib-0043] Zigmond, A. S. , & Snaith, R. P. (1983). The hospital anxiety and depression scale. Acta Psychiatrica Scandinavica, 67(6), 361–370. 10.1111/j.1600-0447.1983.tb09716.x 6880820

